# The clinical and functional outcomes of closed reduction and arthroscopic McLaughlin procedure in patients with neglected locked posterior shoulder dislocation

**DOI:** 10.1051/sicotj/2024050

**Published:** 2024-12-03

**Authors:** Wessam Fakhery Ebied, Ahmed Saeed Younis, Mohamed Amr Hemida, Ahmed H. Khater, Yahia Haroun

**Affiliations:** 1 Orthopedic Department, Faculty of Medicine, Ain Shams University 38 Abbassia, next to the Al-Nour Mosque 11566 Cairo Egypt; 2 Orthopedic Surgery, Ain Shams University 38 Abbassia, next to the Al-Nour Mosque 11566 Cairo Egypt

**Keywords:** McLaughlin’s Procedure, Reverse Hill-Sachs, Locked posterior shoulder dislocation

## Abstract

*Introduction*: Posterior shoulder dislocation with a reverse Hill-Sachs lesion is a rare and complex injury, requiring specialized treatment due to the difficulty in diagnosis, reduction, and addressing both sides of the pathology to reduce the potential for recurrent dislocation. *Purpose*: To evaluate the clinical and functional outcomes of closed reduction and arthroscopic McLaughlin procedure with posterior labral repair in patients with neglected locked posterior shoulder dislocation for less than 12 weeks. *Methods*: A prospective study was conducted at university hospitals, managing 15 patients with neglected locked posterior shoulder dislocation for less than 12 weeks and concomitant engaging reverse Hill-Sachs lesions of less than 40% of the humeral articular surface. They were treated with closed reduction and arthroscopic McLaughlin procedure with posterior labral repair. Patients’ assessments included shoulder range of motion, pain levels using the visual analog scale (VAS) score, and functional outcome using the Oxford instability score and the University of California Los Angeles Shoulder Scale (UCLA) with at least 2 years of postoperative follow-up. *Results*: All 15 patients reported no recurrent dislocation and restored shoulder motion at the final follow-up. External rotation significantly improved from 0° to a mean of 65° in adduction, at 90° of abduction, the respective measurement was 85° (*p* < 0.01). Active forward flexion increased from 35° to 145° (*p* < 0.01). UCLA and Oxford instability scores Showed marked improvement (*p* < 0.01). *Conclusion*: Closed reduction and arthroscopic McLaughlin procedure with posterior labral repair is a safe and effective way for managing patients with locked neglected posterior shoulder dislocations that have been neglected for less than 12 weeks with engaging reverse Hill-Sachs lesion defect, less than 40% of the humeral head.

## Introduction

The glenohumeral joint is the most common dislocated joint in the human body due to its bony anatomy [1]. Posterior shoulder dislocation with a reverse Hill-Sachs lesion is a rare and complex injury, requiring specialized treatment due to the difficulty in diagnosis, reduction, and addressing both sides of the pathology to reduce the potential for recurrent dislocation [2–4]. Posterior shoulder dislocation accounts for 2–5% of all shoulder dislocations and is usually caused by high-energy traumatic mechanisms such as convulsions or electrocution [5]. This type of dislocation is often missed during the initial evaluation, leading to misdiagnosis in 50–79% of cases [3]. Various treatment options exist for managing posterior shoulder dislocations ranging from observation to shoulder arthroplasty, depending on factors such as the patient’s functional status, the duration between the injury and the diagnosis, as well as any associated bone defects in the glenoid and humeral head [6]. Traumatic posterior shoulder dislocations typically involve a reverse Hill-Sachs bone defect, causing a compression fracture anteriorly on the head of the humerus that can engage on the posterior rim of the glenoid causing a locked posterior dislocation [7]. There are several non-anatomic and anatomic surgical techniques to manage humeral head bone defects and restore glenohumeral joint stability [3, 6–11]. McLaughlin was the first to describe the open reconstruction of a reversed Hill-Sachs bone defect by the transfer of the subscapularis tendon [12]. Several surgical techniques for performing this procedure both open and arthroscopic have been described [9, 13–20].

There is no gold standard treatment for locked posterior shoulder dislocation [10, 11, 21]. This prospective study aimed to assess the clinical and functional outcomes of the closed reduction and arthroscopic McLaughlin with posterior labral repair, in managing patients with neglected locked posterior shoulder dislocations for less than 12 weeks. We hypothesized that an all-arthroscopic technique is a safe and efficient approach that could improve shoulder function and restore joint stability in treating these complex cases.

## Materials and methods

We conducted a prospective study to assess the clinical outcomes of patients with neglected locked posterior shoulder dislocation lasting less than 12 weeks, who underwent closed reduction and arthroscopic McLaughlin’s procedure with posterior labral repair. These patients were treated at university hospitals from January 2016 to December 2021.

We included fifteen patients aged between 20 and 60 years old with locked posterior shoulder dislocation with a Hill-Sachs < 40% of the head of the humerus based on the computed tomography (CT) scan performed preoperatively. We excluded patients with acute non-locked dislocations, atraumatic dislocations, patients with fracture dislocation, glenoid bone loss, reverse Hill-Sachs exceeding 40% of the humeral head, glenohumeral osteoarthritis, neurological injury, posterior dislocations missed for more than 12 weeks and patients with an irreducible dislocation by a closed method.

**Figure 1:**
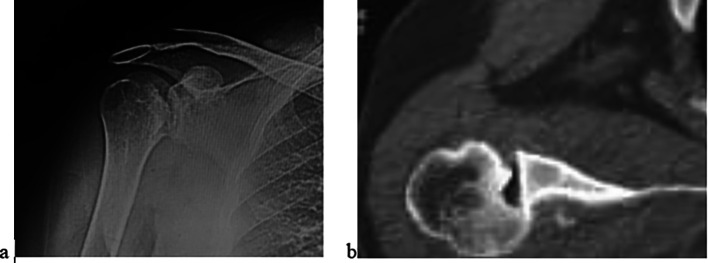
Preoperative imaging of right shoulder with locked posterior shoulder dislocation and reverse Hill-Sachs. (a) Anteroposterior X-ray radiograph and (b) Axial CT scan cut.

All patients had a preoperative clinical shoulder examination, including range of motion (ROM) measurement and visual analog scale (VAS) for pain [22]. The functional status was evaluated using the University of California Los Angeles Shoulder Scale (UCLA) [23, 24] and Oxford instability scores [24, 25]. All patients had a shoulder CT scan with the measurement of the Hill-Sachs lesion percentage from the humeral head (Figure 1). The size of the Hill-Sachs defect was measured on a CT scan at the greatest head diameter and was expressed as a percentage of the articular surface [26]. All cases were related to seizures and the reason for the delayed diagnosis of the dislocation was misdiagnosis and delayed presentation of the patients. A neurologist reviewed the patients pre- and post-operatively to ensure optimal control of the underlying seizures.

### Operative technique and rehabilitation protocol

All patients had general anesthesia and received an ultrasound-guided brachial plexus block. A trial of closed reduction was carried out under an image intensifier. After a successful closed reduction, standard glenohumeral arthroscopy was performed using a 30° scope through a posterior portal. An anterior portal was established using an outside-in technique with shoulder cannula insertion. An anterolateral trans-cuff portal was also established using an outside-in technique with shoulder cannula insertion. The reversed Hill-Sachs defect was prepared using a rasp while viewing from the anterolateral portal. One or two double-loaded 5 mm corkscrew anchors (Arthrex^®^, Naples, FL) were inserted via the anterior portal into the defect. Sutures were passed through the subscapularis tendon using tissue penetrators, and knot tying was done using a knot pusher. Attention was then directed to address the posterior labrum tear. Two all-suture or 2.8 mm titanium anchors (Arthrex^®^, Naples, FL) were inserted through a posterolateral portal into the posterior glenoid. Sutures were passed into the posterior labrum, and knots were tied using a knot pusher. Finally, the portals were closed, and the arm was placed in an abduction pillow. Immediately after surgery, shoulder shrugging, and active elbow, forearm, hand, and wrist ROM were encouraged. The extremity was placed in an abduction pillow for 4 weeks and patients were allowed to do passive and assisted active forward flexion as tolerated. From 5 to 10 weeks, the patients commenced an active ROM as comfort allowed. From 11 to 16 weeks, the patients were allowed full ROM and strengthening exercises.

### Patient evaluation

All patients attended routine regular follow-up appointments at the specialized shoulder clinic. They returned after 2 weeks for stitch removal and subsequent follow-ups to assess clinical improvement and rule out any surgery-related complications. At the 2-year follow-up, we evaluated the recurrence rate, shoulder ROM, pain using VAS, and the patient’s functional outcomes using the Oxford and UCLA Shoulder Scale.

### Data analysis

Data were analyzed by the Statistical Package for Social Science, version 20. A paired *t*-test was used to compare two paired groups using quantitative data and parametric distribution. Repeated Measure Analysis of Variance (ANOVA) was used to compare more than two paired groups with quantitative data and parametric distribution.

## Results

Fifteen patients met the inclusion criteria, and their demographic characteristics are illustrated in (Table 1).

**Table 1:** Demographic data and characteristics of the studied patients.

**Age at surgery**	
Mean ± SD	30 ± 7.22
Range	25–45
**Sex**	
Male	10
Female	5
**Side**	
Right	8
Left	7
**Duration of symptoms in weeks**	
Mean ± SD	5.60 ± 3.02
Range	2–12

The duration of dislocation ranged from 2 to 12 weeks, with a mean of 5.6 weeks. The mean interval between the traumatic event and the operation was 5 weeks (2–12 weeks).

Clinically, all patients regained shoulder motion, including external rotation at 0° and 90° of abduction, as well as forward flexion (*p* < 0.01). Additionally, all patients experienced a substantial decrease in VAS pain score (*p* < 0.01).

They also demonstrated significant functional improvement in UCLA and Oxford instability scores (*p* < 0.01).

**Figure 2:**
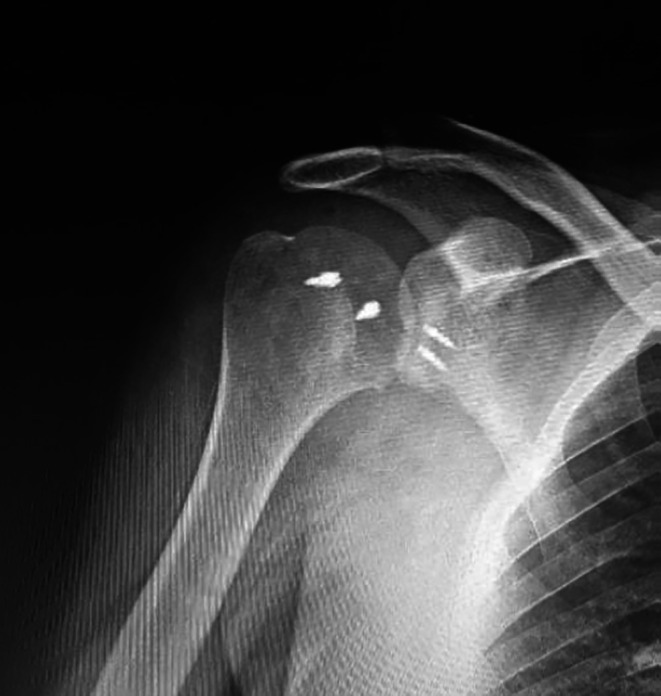
Postoperative radiograph of right shoulder after closed reduction and arthroscopic Mclaughlin procedure with posterior labral repair.

**Figure 3:**
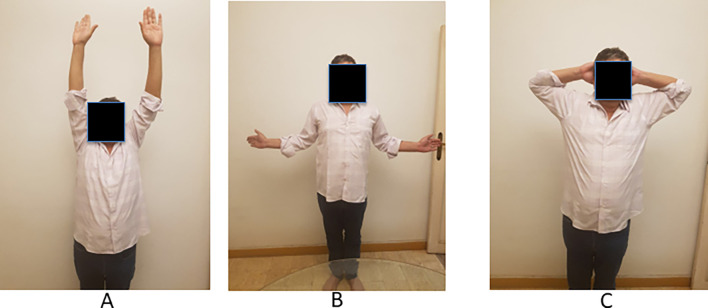
Left shoulder ROM at 2-year final follow-up after closed reduction and arthroscopic Mclaughlin procedure with posterior labral repair. (a) active anterior elevation, (b) active external rotation elbow at side, (c) active external rotation in abduction.

All patients resumed their previous daily activities without any recurrence of dislocation at the 2-year follow-up (Table 2, Figures 2 and 3). These results imply that the proposed procedure could effectively restore the patient’s baseline activity levels.

**Table 2:** Comparison of shoulder ROM and function between preoperative and postoperatively values at the final follow-up.

Parameter	Preoperative	Latest follow-up	*P-*value
	Mean ± SD	Mean ± SD	
	Range	Range	
Forward flexion	34.33 ± 13.48	145.33 ± 19.22	<0.001
	15–50	110–170	
External rotation in adduction	2.00 ± 4.14	63.33 ± 9.76	<0.001
	0–10	40–80	
External rotation in 90 abduction		82.67 ± 7.04	<0.001
		70–90	
UCLA	10.73 ± 7.07	32.47 ± 1.19	<0.001
	4–22	29–34	
Oxford instability score	6.20 ± 4.87	43.47 ± 2.10	<0.001
	3–16	40–48	
VAS	5.07 ± 1.16	2.47 ± 0.52	<0.001

## Discussion

Locked posterior shoulder dislocation with a reverse Hill-Sachs lesion presents a challenge because of its rarity, difficulty and delay in diagnosis, reduction challenge, and different treatment options without a gold standard [2, 10, 11, 21, 27]. In the present study, all patients achieved excellent clinical and functional outcomes at the final follow-up with significant improvement in pain scores, ROM, and functional scores. None of the patients developed recurrent episodes of posterior dislocation and all were satisfied with the outcomes. All returned to their previous activities of daily living.

The limitations of our study were related to the rarity of the injury and the absence of a control group for comparison. Nonetheless, this study to our knowledge is the first to prospectively study such a rare and complex injury with strict inclusion criteria using an all-arthroscopic technique and follow-up for at least 2 years.

Traumatic Posterior shoulder dislocations are rare complex injuries representing 2–5% of all traumatic shoulder dislocations and they are missed in up to 60–79% of cases [28]. Traditionally, they are related to high-energy trauma or seizures [3, 5]. The diagnosis is quite challenging and requires a high clinical suspicion, proper clinical examination, and adequate radiographs [7].

A thorough clinical examination usually shows anterior shoulder flattening, a prominent coracoid process, and severe limitation in external rotation and abduction [5]. True anteroposterior view shows a light bulb sign while the axillary or scapular Y views can show the posteriorly dislocated humeral head [5, 29]. A CT scan can show the dislocation, the extent of the reversed Hill-Sachs defect, and any associated fractures [8, 10, 11, 30].

Decision-making for managing locked posterior dislocation as well as prognosis depends on the size of the reversed Hill-Sachs lesion, duration of the dislocation and patient factors such as; age, comorbidities, and activity levels [10, 11]. Management options include conservative and surgical procedures such as; subscapularis tendon transfer (McLaughlin) [2], lesser tuberosity transfer (Hawkins modified McLaughlin) [13], humeral head reconstruction using allograft or autograft [27, 31], rotation humeral osteotomy [32, 33] and arthroplasty [34] (Table 3).

**Table 3:** Different methods of management of posterior shoulder instability [35].

Procedure	Consideration
**Soft tissue**	
Reverse Bankart repair (open or arthroscopic)	Often performed in combination with an arthroscopic capsular plication, posterior-inferior capsular shift, or reverse Putti-Platt.
Arthroscopic capsular plication	Performed on patients with isolated unidirectional posterior instability without a true labral tear.
Open posterior-inferior capsular shift	The surgical option for patients with posterior-inferior subluxation with no anterior component and a functionally intact rotator interval Reverse Putti-Platt often reduces the range of motion and is thus generally not recommended for athletes requiring full range of motion.
Thermal capsulorrhaphy	Not recommended because of high recurrence rates.
**Osseous**	
Posterior bone block or posterior wedge osteotomy	Generally indicated for patients presenting with a failed capsular plication, glenoid hypoplasia, increased glenoid retroversion, or an osteochondral fracture of the glenoid cavity versus posterior glenoid bone loss.
McLaughlin’s procedure or Neer’s modification of McLaughlin’s	Performed on patients with locked posterior shoulder dislocation resulting from reverse Hill-Sachs lesions encompassing 25–50% of the humeral head.
Humeral head allograft	An alternative option to McLaughlin’s or Neer’s procedures based on the surgeon’s preference/experience; our preference is the most anatomic way to reconstruct large engaging reverse Hill-Sachs lesions.

Multiple arthroscopic techniques have been described to manage the reverse Hill-Sachs in patients with posterior shoulder dislocations [14, 15]. Krackhardt et al. [17] described subscapularis tendon detachment and mobilization to fill the humeral bone defect and a similar arthroscopic technique to transfer the middle glenohumeral ligament was described by Duey and Burkhart [36]. Martetschläger et al. [14] described a modified version of the original McLaughlin technique to arthroscopically transfer the subscapularis tendon into the defect without detachment. Others have described an arthroscopic technique for reverse remplissage using suture anchors without subscapularis detachment [15, 18, 20]. However, these technical notes did not study the clinical and functional outcomes.

Brilakis et al. [19] conducted the first retrospective study on 10 patients to evaluate the outcomes of the arthroscopic modified McLaughlin procedure using shoulder ROM, UCLA, and Oxford instability score. Their results concurred with ours, with improved outcomes and no recurrence. In contrast to our study, they excluded patients with epilepsy, the mean age was higher at 50 years, the mean duration of dislocation was longer at approximately 2.7 months and ranged from 0.5 to 10 months.

Ippolito et al. [37] conducted a retrospective study to compare the management of ten patients’ irreducible locked posterior dislocation of the shoulder associated with reverse Hill–Sachs using open Neer modification of the McLaughlin procedure and all-arthroscopic McLaughlin procedure. Both groups did well with the surgery without differences in the functional outcomes. However, the sample size was too small and patients’ allocation to each group relied on surgeon preference.

Romano et al. [38] were the first to study arthroscopic reduction then arthroscopic McLaughlin and posterior labral repair in 12 patients with locked posterior shoulder dislocation with reverse Hill-Sachs defect of less than 30%. They found excellent improvement in the clinical and functional outcomes in all patients, with no recurrence, and all patients were satisfied.

Our arthroscopic technique is minimally invasive, quite simple, and reproducible for shoulder surgeons accustomed to performing arthroscopic Bankart repair and remplissage. It uses a standard shoulder arthroscopy setup, instruments, portals, and suture anchors. It can be done in either a beach chair or lateral decubitus position according to surgical preference. However, additional studies are needed to evaluate the efficacy of such a technique about shoulder stabilization and functional outcomes.

## Conclusion

Closed reduction and arthroscopic McLaughlin for patients with neglected locked posterior shoulder dislocation for less than 12 weeks is an effective and safe way of managing such a complex and rare injury, without the morbidity of alternative open surgical options.

## Data Availability

All data and materials are available when required.
